# The unique role of lexical accessibility in predicting kindergarten emergent literacy

**DOI:** 10.1007/s11145-015-9614-8

**Published:** 2016-01-27

**Authors:** Ludo Verhoeven, Jan van Leeuwe, Rosemarie Irausquin, Eliane Segers

**Affiliations:** Faculty of Social Sciences, Behavioural Science Institute, Radboud University Nijmegen, P.O. Box 9044, 6500 KD Nijmegen, The Netherlands

**Keywords:** Emergent literacy, Phonological awareness, Letter knowledge, Kindergarten

## Abstract

The goal of this longitudinal study was to examine how lexical quality predicts the emergence of literacy abilities in 169 Dutch kindergarten children before formal reading instruction has started. At the beginning of the school year, a battery of precursor measures associated with lexical quality was related to the emergence of letter knowledge and word decoding. Confirmatory factor analysis evidenced five domains related to lexical quality, i.e., vocabulary, phonological coding, phonological awareness, lexical retrieval and phonological working memory. Structural equation modeling showed that the development of letter knowledge during the year could be predicted from children’s phonological awareness and lexical retrieval, and the emergence of word decoding from their phonological awareness and letter knowledge. It is concluded that it is primarily the accessibility of phonological representations in the mental lexicon that predicts the emergence of literacy in kindergarten.

## Introduction

Research on emergent literacy has shown that interactive activities, such as storybook reading, communicative writing and language games, help children to get insight into the functions and structure of written language and to discover the written code (see Mol, Bus, & de Jong, 2009). The extent to which preliterate children learn to grasp the written code may be highly dependent on abilities associated with lexical quality: vocabulary breadth and depth (Metsala & Walley, 1998; de Jong & Olson, 2004), phonological decoding (Burgess & Lonigan, 1998), phonological awareness (Goswami, 2000), lexical retrieval (Kim & Petscher, 2011), and verbal working memory (Brunswick, Martin, & Rippon, 2012) all have an impact on the emergence of literacy. Although this lexical quality hypothesis is supported by empirical evidence (Perfetti & Stafura, 2014), the relative importance of these lexical quality abilities on the emergence of literacy is far from clear. In the research so far, no attempt has been made to investigate the contribution of all of these factors of lexical quality on the development of literacy in preliterate children in one and the same design. Therefore, in the present study, it was examined to what extent the development of letter knowledge and word decoding could be predicted from a broad range of lexical quality predictors in kindergarten children in the Netherlands.

In a rich literacy environment, children learn that print carries meaning, that written texts may have various forms and functions, and that ideas can be expressed with (non)conventional writing (see Yaden, Rowe, & MacGillivray, 2000). In the case of alphabetic languages, children learn that words consist of phonemes which can be represented by letters. There is general agreement that in the case of alphabetic writing systems the acquisition of literacy involves the learning of the principles of phonological recoding (Ehri, 2005, 2014; Leinenger, 2014). In the process of understanding written language, children begin with a rough approach of a limited collection of words that have personal meaning to them. Subsequently, they discover the alphabetic principle on the basis of an analysis of familiar words involving their constituent sounds and letters. Phonological recoding can be seen as an inductive learning mechanism on the basis of which children learn to crack the code by mapping letters to sounds (see Share, 1995, 2004), while phonological mediation remains an obligatory component of lexical access which is routinely activated in advanced reading (see Coltheart, Rastle, Perry, Langdon, & Ziegler, 2001; Perfetti & Stafura, 2014). Given the fact that visual word identification consists of connecting a familiar phonological form with an orthographic form in order to address meaning, it can be assumed that lexical quality plays an essential role in children’s early understanding of the alphabetic principle. Exactly how abilities associated with lexical quality in preliterate children can be monitored and in what way they predict the acquisition of literacy before the time formal literacy instruction is started is not clear yet. We investigated five domains of lexical quality abilities which may have an impact on the emergence of literacy.

The first domain is vocabulary. In a context-rich environment, children learn to increase their stock of content words and to refine and narrow down the specific meanings of words. With the gradual increase of the number of words in the mental lexicon, there is a continuous pressure to make finer phonological distinctions to accommodate the efficient storage of words. According to the lexical restructuring hypothesis (Metsala & Walley, 1998), lexical representations start out to be holistic but get refined and better specified over the years. In line with the lexical quality hypothesis, it can be predicted that the breadth and depth of children’s oral vocabularies predict the degree to which words in the mental lexicon are phonologically specified and early literacy can emerge (see Verhoeven, van Leeuwe, & Vermeer, 2011).

The second domain is phonological coding which involves the representation of information about the sound structure of verbal stimuli in memory (Torgeson, Wagner, Rashotte, Burgess, & Hecht, 1997; Perfetti, 1992). It can be assumed that the quality of a word representation is dependent on its precision, or its degree of specification. Partially specified representations lack the potentially available word-specific information which may set the stage for the discovery of the alphabetic principle. The importance of highly specified phonological representations for early literacy development has been demonstrated in the early work by Shankweiler and Liberman (1989) and Fowler (1991). A key factor in phonological coding is speech perception. As children are exposed to a continuous speech stream from the environment, they must parse the incoming acoustic signal into consistent, replicable chunks that will come to represent the phonemes (cf. Kuhl 2011). It has been found that a lack of full auditory discrimination of speech sounds may hamper the onset of the inductive learning mechanism which is able to acquire new letter names and to form words with them (Reed, 1989; Stackhouse, 2000). Another important aspect of phonological coding concerns phonological sensitivity, or the relative specificity with which a lexical item is represented. According to Elbro (1996), phonological sensitivity can be seen as a function of the number of distinctive features of the representation being encoded in the mental lexicon. Elbro, Borstrom, and Petersen (1998) found this measure to be a predictor of the emergence of letter knowledge and the development of phonological recoding skills in later reading. Phonological sensitivity can be measured by tapping children’s (masked) word recognition (Munson, 2001), or (non)word repetition (Baird, Slonims, Simonoff, & Dworzynski, 2011), although the latter is also considered to be related to verbal working memory (Gathercole, 2006).

The third domain is phonological awareness—the awareness of speech sounds in a word (cf. Wagner & Torgesen, 1987; Swanson, 2003). There is abundant research evidence showing that phonological awareness is needed for the child to learn that words consist of phonemes and that these phonemes can be represented by graphemes (cf. Goswami, 2001; Anthony & Lonigan, 2004; Lonigan, Burgess, & Anthony, 2000). Phonological awareness requires children to reflect consciously on the phonological segments of spoken words and to manipulate them in a systematic way. As such, phonological awareness depends on the capacity to focus attention on the perceptual representations of speech (Mann, 1991). It can be assessed by tasks measuring segmentation, blending, and manipulation of speech sounds (Yopp, 1988; Vloedgraven & Verhoeven, 2007). Research shows the development of phonological awareness to progress from the syllable level and the onset-rime level to the phoneme level (cf. Shankweiler & Liberman, 1989; Lonigan, 2006). Relatively easy for children is sensitivity to rhyme (Vloedgraven & Verhoeven, 2009). More difficult is phonemic awareness which concerns the awareness of phonemes, the speech sounds or units of sound that are used to build spoken words and to distinguish meanings (cf. Nagy & Scott, 2000; Goswami, 2000). Numerous studies have shown a substantial relation between measures of phonemic awareness administered to five-year olds and early literacy measures in kindergarten and first grade (cf. Swanson, Trainin, Necoechea, & Hammill, 2003; Moll et al., 2014a, b).

The fourth domain of lexical quality is the capacity to retrieve stored lexical representations from memory. For any kind of orthographic processing, it is important that visual representations can be fast retrieved from memory. This capacity can be assessed by rapid automatized naming (RAN) tasks measuring the rate at which one can name a randomly repeatedly presented limited set of visual stimuli, such as pictures, colors, letters or numbers. RAN tasks require the fast phonological access to stored visual representations (see Parrila, Kirby, & McQuarrie, 2004; Vaessen, Gerretsen, & Blomert, 2009). In the literature, a systematic relation between RAN scores and early reading fluency measures has been evidenced (see Lervag & Hulme, 2009; Moll et al., 2014a, b) which can be explained from the fact that both capacities involve direct access to previously stored visual stimuli (Decker, Roberts, & Englund, 2013) as well as visual-verbal integration (Kirby, Georgiou, Martinussen, & Parrila, 2010).

The fifth and final domain of lexical quality is verbal working memory (WM). Although WM has been conceptualized in several theoretical models (Courage & Cowan, 2009), the most applied model in previous research is Baddeley’s multicomponent WM model (Baddeley, 1986, 2012), consisting of a central executive linked with three subsystems: phonological loop, visuospatial sketchpad and episodic buffer. The phonological loop and visuospatial sketchpad are slave-systems, responsible for the temporary storage of verbal and visuospatial information respectively. The central executive is responsible for the coordination and control of different activities in WM. Phonological loop and central executive which are commonly assessed by means of a forward and backward digit span task have indeed shown to be relevant for the emergence of letter knowledge (cf. de Jong & Olson, 2004; Silva, Faísca, Ingvar, Petersson, & Reis, 2012), the assembling of phonological codes (Berninger et al., 2006) and the development of word recognition (e.g., Alloway, Gathercole, Adams, Eaglen, & Lamont, 2005).

In conclusion, the literature shows that various domains related to lexical quality abilities may have an effect on the emergence of literacy: vocabulary size, rapid naming, phonological coding, phonological awareness and verbal working memory. The problem is, however, threefold. First of all, previous research has focused mainly on the influence of these factors on reading and writing in primary school. The impact of lexical quality abilities on the emergence of literacy, i.e., before formal reading instruction in school has started, has received only scant attention. Second, in the studies conducted so far, no attempt has been made to relate the impact of predictor measures from the five lexical quality domains on early literacy in one and the same design. Thus, the relative contribution of vocabulary size, rapid naming, phonological coding, phonological awareness and verbal working memory to emergent literacy has not yet been evaluated. Finally, previous studies show shortcomings in measuring lexical quality domains. Predictor variables have often been operationalized by only single measures. Insofar multiple measures have been used, they were not validated by means of factor analytic procedures.

In the present study, an attempt was made to examine the role of lexical quality on emergent literacy in 169 kindergartners in the Netherlands. At the beginning of the second kindergarten year (age 5), a broad range of tasks were administered to assess children’s vocabulary, phonological coding, phonological awareness, lexical retrieval and verbal working memory. For each of these domains, we included at least two measures. For vocabulary, we focused on vocabulary breadth and depth, for phonological coding on speech perception and phonological sensitivity, for phonological awareness on differential task complexities, for lexical retrieval on rapid naming and name generation speed, and for verbal working memory on phonological loop and executive functioning. By means of confirmative factor analysis, an attempt was made to find empirical evidence for the constructs we intended to measure. To examine the emergence of literacy, we measured children’s knowledge of grapheme–phoneme relations at the beginning and at the end of the year, and word decoding at the end of the year. In order to find out to what extent the emergence of literacy could be predicted from lexical quality precursors, the latent variables of vocabulary, lexical retrieval, phonological coding, phonological awareness and verbal working memory achievement predict children’s letter knowledge at age 5 were related to (1) children’s letter knowledge at the same moment of measurement (age 5) and (2) their letter knowledge and word decoding ability one year later (age 6).

## Method

### Participants

A total of 169 native Dutch children (98 boys, 71 girls) of middle socio-economic status took part in the study. They were recruited from 7 regular primary schools (including kindergarten) in the Netherlands. Dutch children normally enter elementary school by the age of 4 and in none of the cases were there any reports on language impairment or hearing loss. During the first 2 years, children follow a kindergarten curriculum. The focus is on informal settings in which children are immersed in storybook reading and language games, whereas emergent literacy activities in a playful setting are also part of the curriculum. The parents of the children had given approval for participation by written consent. At the start of the study, the children were at the beginning of their second year of kindergarten and their average age was 5 years 3 months (SD = 3.70 months).

### Instruments

#### Precursor measures

As precursor measures, instruments were used to assess vocabulary breadth and depth, phonological coding abilities, phonological awareness, lexical retrieval, and working memory.

#### Vocabulary

##### Receptive vocabulary (RV)

The Passive Vocabulary of the Dutch Language Test for Children (Verhoeven & Vermeer, 2001) was administered to measure receptive vocabulary breadth. In this task, children were presented with 96 items which are representative of the words used by children in the early primary grades, each of which contained four pictures along with an orally presented word matching with one of the pictures. The total number of correctly matched words comprised the score on this task. Cronbach’s alpha was 0.97 which points to a high reliability of the test.

##### Productive vocabulary (PV)

To measure productive vocabulary depth, the Productive Vocabulary task of the Dutch Language Test for Children (Verhoeven & Vermeer, 2001) was administered. This task contained 60 pictures to be named by the child with the number of correctly named words comprising the score. Reliability of test was high with a Cronbach’s alpha of 0.91.

#### Phonological coding measures

##### Phonological distinctness (PD)

This test was based on a measure proposed by Elbro et al. (1998) which was designed to elicit the most distinct pronunciation of words. The task consists of 23 polysyllabic high frequency words in which certain syllables have been reduced or omitted. In each word one or two unstressed syllables were omitted. Additionally another syllable in the same word could be reduced. A hand-held puppet was shown to the child. Then the child was told that the puppet wanted to learn to pronounce words correctly and that it needed some help from the child. For each item the experimenter showed a picture and pronounced the corresponding sound incompletely, e.g., *ofan* with the picture of an elephant (Dutch: *olifant*). The child was asked to complete the word and to sound it out loudly for the puppet. The experimenter then repeated the word until the child made no further corrections. There were three practice items on this task. The total number of words sounded out correctly constituted the test score (PD1). As an additional measure the number of syllable reductions was computed (PD2) as a sign of difficulty in sounding out the correct word form. The test showed reasonable reliability (Cronbach’s alpha of 0.72).

##### Auditory discrimination (AD)

This task is a subtest of the standardized *Dutch language test for children* (Verhoeven & Vermeer, 2001). In the task the child was presented 50 minimal word pairs in which the words were the same or different in one constituent phoneme. For each item the child was asked to indicate whether word pairs were same or different. There were two practice items on this task. The number of correct answers counted as the score on this task. The reliability of the test was high with Cronbach’s alpha being 0.90.

##### Nonword repetition (NWR)

In this task the child was asked to repeated nonwords spoken out by the experimenter. The task consisted of three practice items of one syllable and 22 test items varying in length and syllabic complexity. The number of correctly repeated nonwords comprised the score on this task. The test showed good reliability with Cronbach’s alpha being 0.83.

##### Word closure (WC)

This task is a subtest of the standardized *Language test for children* (van Bon & Hoekstra  1982). It consists of five practice items and 29 test items. In each item a polysyllabic word was presented auditorily from audiotape with one to three consonants being deleted, e.g., *radio* was presented as *ra*-*io*. Each word pattern was presented twice before the child was asked to say the word. The total score was the number of correctly produced words. Reliability was good with a Cronbach’s alpha of 0.81.

##### Masked word repetition (MWR)

In this task the child was given 48 monosyllabic words one-by-one to the left or the right ear with a −2 or −5 dB speech to noise ratio. The child had to say the word (s)he had heard. There were four practice items on this task. The total number correctly produced words comprised the score on this task. Reliability was reasonable with Cronbach’s alpha being 0.79.

#### Phonological awareness measures

##### Receptive rhyme (RR)

In this task the experimenter presented orally 10 pairs of monosyllabic words to the child, half of which had corresponding rimes. For each word pair the child was asked whether the words rhymed or not. There were three practice items on this task. The number of correctly answered items constituted the score on this task. Reliability was reasonable with a Cronbach’s alpha of 0.79.

##### Productive rhyme (PR)

In this task the experimenter presented 10 CVC words one by one and asked the child to say a rhyming word. An example was given along with three practice items. The score on this task was the number of correct rhymes produced by the child. Reliability was reasonable with a Cronbach’s alpha of 0.77.

##### Phoneme segmentation (PS)

In this task, the child was asked to segment words in their constituent phonemes. This task consists of three practice items (CVC words) and 30 test items (10 CVC, 10 CCVC and 10 CVCC words). The number of correct answers comprised the score on this task. Reliability was reasonable with a Cronbach’s alpha of 0.74.

##### Word blending (WB)

In this task, the experimenter presented the phonemes of individual words one-by-one and asked the child which word could be sounded out if the sounds were ‘glued together’. This task consists of three practice items (CVC words) and 30 test items (10 CVC, 10 CCVC and 10 CVCC words). The number of correct answers comprised the score on this task. Reliability was reasonable with a Cronbach’s alpha of 0.80.

##### Initial phoneme isolation (IP)

In this task, individual words were presented to the child with the question to isolate the first sound of the word. After three practice items of CVC words, a series of 10 test items of this word type was given. In addition, another set of three practice items of CCVC words was given along with 10 test items of this word type. The score on this task was the total number of correctly answered items. Reliability was reasonable with a Cronbach’s alpha of 0.71.

##### Final phoneme isolation (FP)

In this task, individual words were presented to the child with the question to isolate the final sound of the word. After three practice items of CVC words, a series of 10 test items of this word type was given. In addition, another set of three practice items of CVCC words was given along with 10 test items of this word type. The score on this task was the total number of correctly answered items. Reliability was reasonable with a Cronbach’s alpha of 0.73.

##### Phoneme deletion (DEL)

This task asked from the child to delete the initial or final sound in monosyllabic words. The tasks consisted of four series of 10 test items, each preceded by three practice items: initial CVC, initial CCVC, final CVC and final CVCC phoneme deletion. The score on this task was the total number of correctly answered items. Reliability was reasonable with a Cronbach’s alpha of 0.70.

#### Lexical retrieval measures

##### Rapid naming (RAN)

Children were presented with a card on which five high-frequency pictures were displayed in rows with the instruction to name the pictures accurately and fast. The score on this task was the total number of correctly named pictures in 1 min. Reliability was high with a Cronbach’s alpha of 0.83.

##### Word naming (WN)

Children were asked to name as many words as possible with a specific beginning consonant in 20 s. Nine different consonants were introduced and the total number of correctly named words comprised the children’s score on this task. Reliability was reasonable with a Cronbach’s alpha of 0.79.

#### Working memory

##### Digit span (DS)

To measure differential aspects of working memory we used the WISC subtest Digit Span. Both the recall of series of digits in forward order (Digit Span Forward, DSF) and the recall of series of digits in backward order (Digit Span Backward, DSB) was measured with the number of correctly reproduced series of digits as test scores. Reliability of the task is good with a Cronbach’s alpha of 0.87.

#### Criterion measures

##### Grapheme–phoneme correspondences (GPC)

To measure children’s letter knowledge, children were confronted with a standardized test consisting of card displaying all 34 Dutch graphemes to be read out loud (Verhoeven, 1995). The number of correctly named grapheme–phoneme correspondences comprised the score on this task.

##### Word decoding (WD)

To measure children’s word decoding, the first card of the standardized Three-minutes-test (Verhoeven, 1995) was administered. This card contained orthographic Dutch CVC words and the child was asked to name as many words as possible in 1 min.

### Procedure

At the start of the study the children had just entered their second kindergarten year. The first testing (T1) took place at the beginning of the school year. The second testing (T2) was at the end of the school year. Graduate students administered the tests in a quiet room at school.

The data were analyzed in three steps. First, the means and standard deviations were computed for all tests, and the progress in knowledge of grapheme–phoneme correspondences (GPC) was tested for significance. Second, the initial scores on the lexical quality measures of Time 1 were submitted to confirmatory factor analysis using varimax rotation with the help of the computer program AMOS. Third, we conducted covariance structure analysis with the help of the same program in order to examine the relationships between the precursor measures of vocabulary, phonological coding, phonological awareness, lexical retrieval, and working memory, on the one hand, and literacy abilities (i.e., grapheme–phoneme knowledge development and word decoding), on the other hand. The goodness of fit of estimated models was assessed by five indices: χ^2^ with corresponding degrees of freedom and *p* value, Adjusted Goodness of Fit Index (AGFI), Normed Fit Index (NFI), Root Mean Square Error of Approximation (RMSEA), and Standardized Root Mean Square Residual (SRMR) (Browne & Cudeck, 1993; Jöreskog & Sorbom, 1996). A model could be viewed acceptable when the ration of χ^2^ to the degrees of freedom was found to be smaller than 2:1, the AGFI and NFI values being higher than 0.80, and the RMSEA lower than 0.08 (Hu & Bentler, 1999).

## Results

### Descriptive statistics

In Table 1 the means and standard deviations for all of the tests administered at the beginning and end of the second year of kindergarten are presented. *T* test showed the differences on Grapheme–Phoneme Correspondences to be significant (*p* < 0.001).

**Table 1:** Means and standard deviations on precursor measures of lexical quality and criterion measures of early literacy

	Time 1		Time 2	
	Mean	SD	Mean	SD
Receptive vocabulary (96)	60.20	14.65	–	–
Productive vocabulary (60)	34.54	7.59	–	–
Phonological distinctness 1 (100)	80.84	13.57	–	–
Phonological distinctness 2 (100)	7.98	4.84	–	–
Auditory discrimination (50)	43.91	6.20	–	–
Nonword repetition (100)	77.12	12.19	–	–
Word closure (29)	17.69	4.65	–	–
Masked word recognition (100)	84.27	9.23	–	–
Receptive rhyme (10)	9.60	0.97	–	–
Productive rhyme (10)	9.42	1.49	–	–
Phoneme segmentation (30)	5.24	8.39	–	–
Word blending (30)	7.22	9.41	–	–
Initial phoneme isolation (20)	9.26	8.14	–	–
Final phoneme isolation (20)	8.10	8.21	–	–
Phoneme deletion (20)	5.13	7.22	–	–
Rapid naming pictures (60)	33.15	9.41	–	–
Rapid naming words	37.51	9.83	–	–
Digit span forward (10)	3.16	0.55	–	–
Digit span backward (10)	2.84	1.14	–	–
Grapheme–phoneme corr. (34)	5.40	6.48	11.22	8.63
Word decoding (30)	–	–	2.12	5.36

### Confirmatory factor analysis

**Fig. 1:**
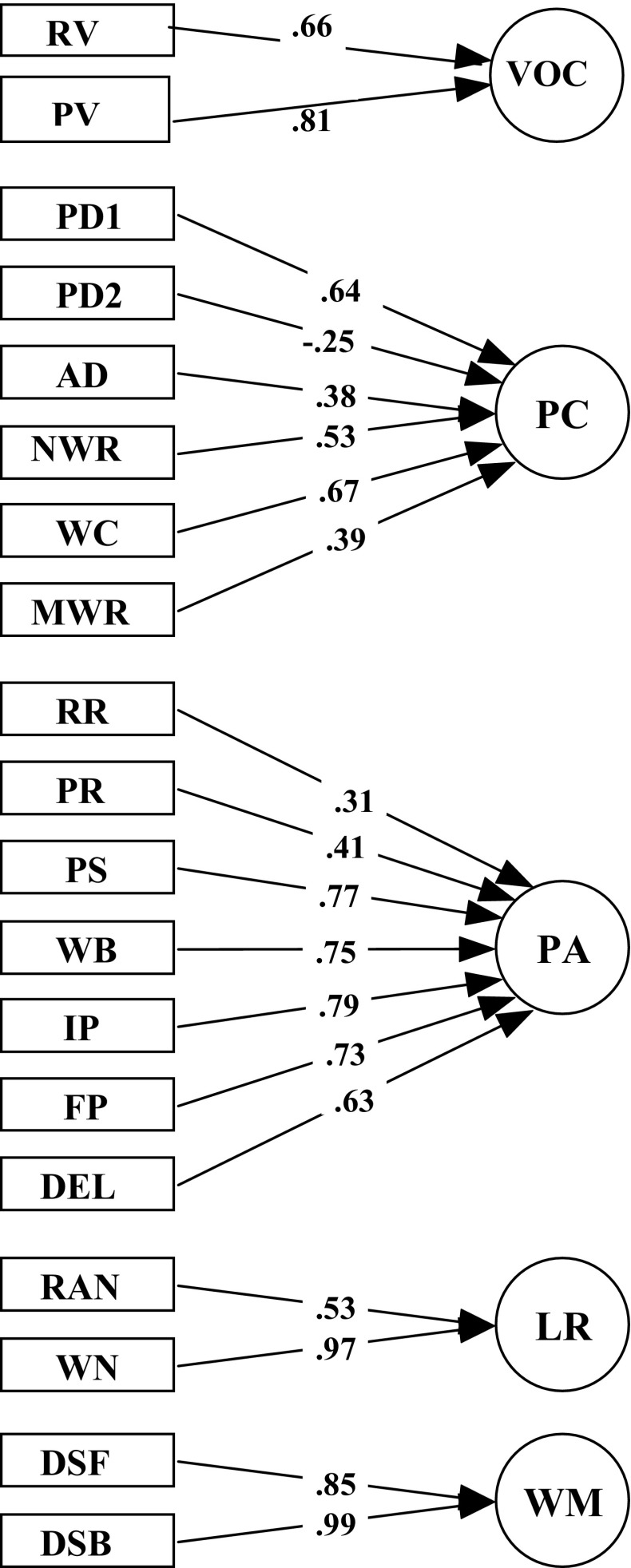
Results of confirmatory factor analysis on the precursor measures yielding the latent factor scores of vocabulary (VOC) from receptive vocabulary (RV) and productive vocabulary (PV); phonological coding (PC) from phonological distinctiveness 1–2 (PD1, PD2), auditory discrimination (AD), non-word repetition (NWR), word closure (WC), and masked word recognition (MWR); phonological awareness (PA) from receptive rhyme (RR), productive rhyme (PR), phoneme segmentation (PS), word blending (WB), initial and final phoneme isolation (IPI, FPI), and phoneme deletion (PD); lexical retrieval (LR) from rapid naming pictures (RAN) and rapid naming words (RNW), and working memory (WM) from digit span forward and backward (DSF, DSB)

Confirmatory factor analysis was conducted to find out to what extent the precursor measures obeyed the predefined structure of factors. Indeed, as is shown in Fig. 1, a five-factor structure gave the best fit to describe precursor measures with factors which could be identified as Vocabulary (VOC), Phonological Coding (PC), Phonological Awareness (PA), Lexical Retrieval (LR), and Working Memory (WM). Alternative models yielded less satisfactory outcomes. All loadings were significant (*p* < 0.01). Model fit of the present factor solution can be called good with Chi square = 195.045, *df* = 140, *p* = 0.001, gfi = 0.892, agfi = 0.854, nfi = 0.842, rmsea = 0.050.

In Table 2, the correlations between the factors are given. It can be seen that there are substantial correlations between the precursor measures, particularly between the factors of phonological coding, on the one hand, and phonological awareness and vocabulary, on the other hand.

**Table 2:** Correlations between latent factor scores of vocabulary (VOC), phonological coding (PC), phonological awareness (PA), lexical retrieval (LR), and working memory (WM)

	VOC	PC	PA	LR	WM
VOC	1				
PC	0.76	1			
PA	0.53	0.68	1		
LR	−0.53	−0.49	−0.40	1	
WM	0.43	0.44	0.42	−0.24	1

### Predictors of letter knowledge and word decoding

**Fig. 2:**
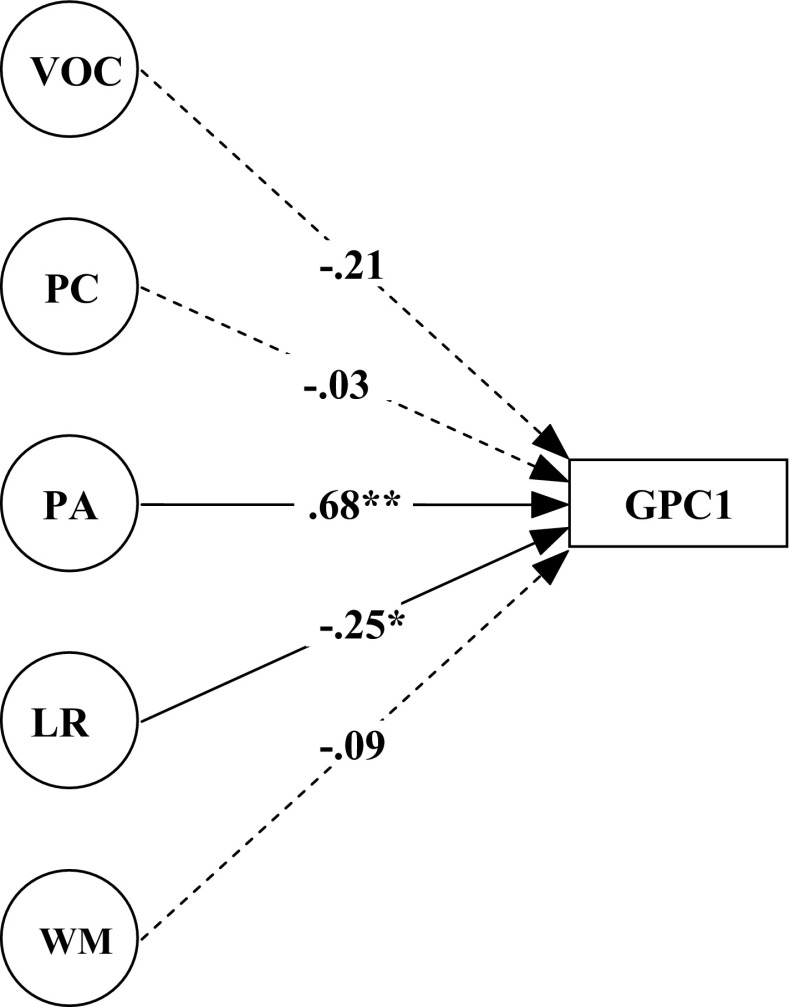
Regression model with grapheme–phoneme correspondences at time 1 (GPC1) being explained from the latent variables of vocabulary (VOC), phonological coding (PC), phonological awareness (PA), lexical retrieval (LR) and working memory (WM)

A series of Structural Equation Modeling (SEM) analyses was carried out in a stepwise manner in order to examine the relationship between proposed components of lexical quality and emergent literacy. First of all, it was examined to what extent the outcomes of GPC1 could be explained from the five types of predictor measures as measured by the latent factors scores of VOC, PC, PA, LR and WM. The resulting model is displayed in Fig. 2. The model fit can be called reasonable with Chi square = 217.996, *df* = 154, *p* = 0.001, gfi = 0.888, agfi = 0.847, nfi = 0.836, and rmsea = 0.051. The model shows that the variation in GPC1 can be explained by the latent variables of PA and LR with 57 % of the variance explained.

**Fig. 3:**
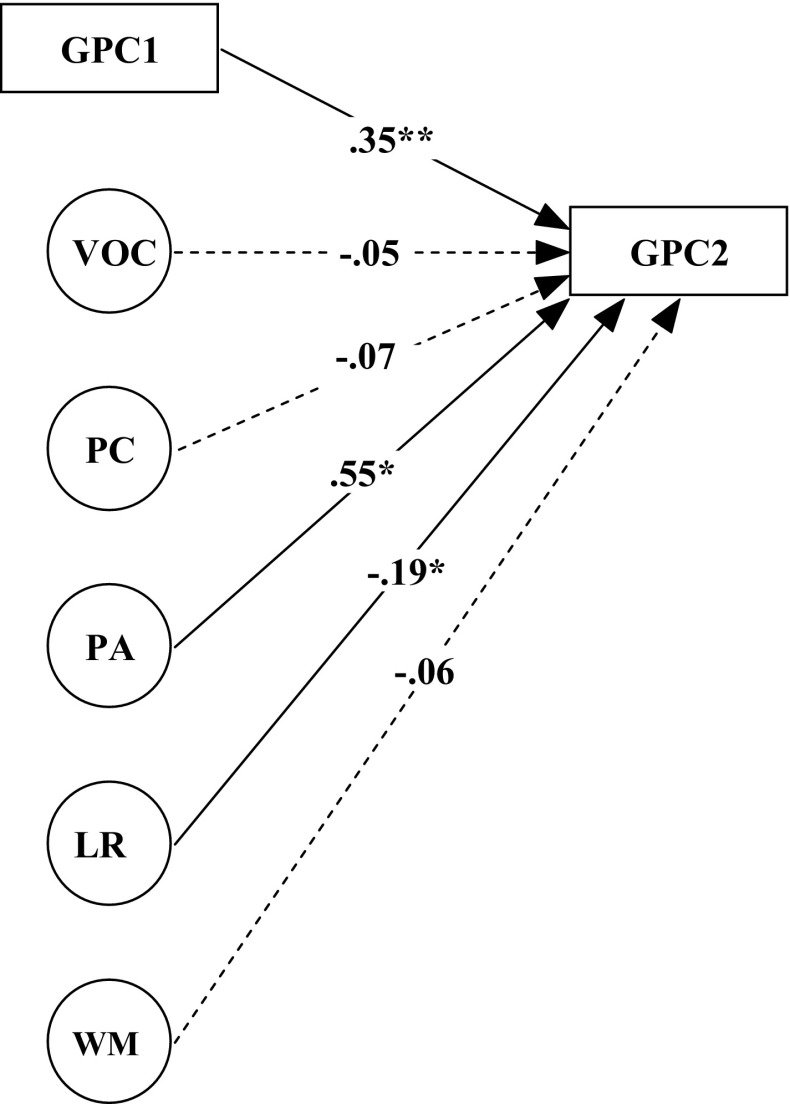
Structural equation model with grapheme-phoneme correspondences at time 2 (GPC2) being explained from the autoregressor GPC1 and the latent variables of vocabulary (VOC), phonological coding (PC), phonological awareness (PA), lexical retrieval (LR) and working memory (WM)

In a subsequent SEM analysis, the prediction of GPC2 by the same latent precursor measures was examined with GPC1 as autoregressor (see Fig. 3). The model fit can again be called reasonable with Chi square = 236.157, *df* = 168, *p* = 0.000, gfi = 0.885, agfi = 0.843, nfi = 0.844, and rmsea = 0.051.

Figure 3 shows that, apart from the autoregressive influx, only the latent variables of Phonological Awareness (PA) and Lexical Retrieving (LR) contribute significantly to the variance of GPC2. The percentage of explained variance in GPC2 is 70.4.

**Fig. 4:**
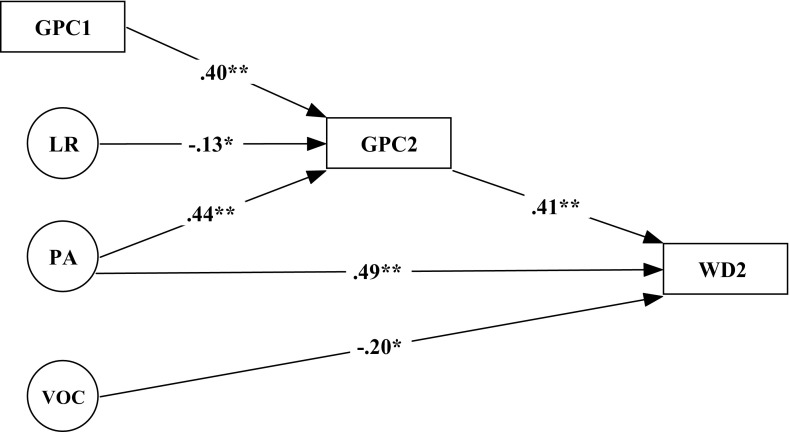
Structural equation model with word decoding 2 (WD2) being explained from both the development of grapheme–phoneme correspondences (GPC) during the year and the latent variables of vocabulary (VOC), phonological coding (PC), phonological awareness (PA), lexical retrieval (LR) and working memory (WM) with no significant contributions evidenced from PC and WM

In a final SEM model, it was examined to what extent the variation in WD2 could be explained from the development of GPC during the year, on the one hand, and the latent precursor measures, on the other hand (see Fig. 4). The model fit can again be called reasonable, given the following indices: Chi square = 97.290, *df* = 65, *p* = 0.006, gfi = 0.919, agfi = 0.869, nfi = 0.911, rmsea = 0.056.

Figure 4 makes it clear that WD2 is predicted by GPC2 and PA, and that GPC2, on its turn, is explained from GPC1, LR and PA. The unexpected negative relation between VOC and WD2 can tentatively be explained from the suppression of VOC by PA, given their strong correlation. The percentage of explained variance in WD2 is 59.3.

## Conclusions and discussion

This study aimed to predict the emergence of literacy skills from children’s lexical quality related abilities in kindergarten before formal literacy has started. Confirmatory factor analysis evidenced five factors representing predefined lexical quality domains: vocabulary, phonological coding, phonological awareness, lexical retrieval, and verbal working memory. It was also shown that children made significant progress in knowledge of grapheme–phoneme correspondences during the year. Making a distinction between the latent precursors as critical domains of lexical abilities, it was questioned which of these precursors would predict the development of letter knowledge and word decoding.

A series of structural equation modeling analyses showed how children’s abilities in the various lexical quality domains related to the emergence of letter knowledge and word decoding. At the onset of the kindergarten year, almost sixty percent of the variation in letter knowledge could significantly be explained from children’s level of phonological awareness and lexical retrieval abilities. It is important to note that the same predictors also prevailed in the prediction of the development of letter knowledge throughout the year: taking children’s initial letter knowledge as autoregressor, phonological awareness and lexical retrieval significantly predicted their level of letter knowledge by the end of the year, explaining more than seventy percent of the variance. Our final analysis concerned the prediction of word decoding by the end of the year, taking into account the progress children made in letter knowledge during the year. The variation in word decoding could be explained from children’s letter knowledge and phonological awareness whereas, on its turn, the variation in letter knowledge could be explained by phonological awareness and lexical retrieval.

The present results highlight the importance of phonological awareness and lexical retrieval in the emergence of early literacy, even after taking into account lexical quality measures in the domains of vocabulary, phonological coding, and verbal working memory. Although the precursor measures were found to be related, it shows that explicit phonological capacities which are involved in phonological awareness and lexical retrieval are the most relevant lexical quality predictors of early literacy before formal reading instruction has started. It is important to note that follow-up processes of learning to read have also been found to be predicted by phonological awareness (cf. Piasta & Wagner, 2010; Ziegler & Goswami, 2005; Melby-Lervag, Halaas Lyster, & Hume, 2012) and lexical retrieval (see Bowers & Wolf, 1993; Logan, Schatschneider, & Wagner, 2011). The latter is often associated with the automated, non-intentional induction of orthographic patterns (cf. Parrila et al., 2004). Neurocognitive support for this claim also comes from a study by Goldberg, Perfetti, and Schneider (2006), showing that the precise timing mechanisms involved in lexical retrieval are highly relevant for the establishing and development of orthographic codes in interaction with phonological codes.

Interestingly, phonological awareness and lexical retrieval can be seen as domains of lexical quality which not so much relate to the specificity of lexical representations or to the level of verbal working memory but rather to the accessibility of lexical representations. Our study shows that even after controlling for precursors relating to the quality of lexical representations, i.e., phonological coding and breadth and depth of vocabulary, as well as verbal working memory, phonological awareness and lexical retrieval predict the development of early literacy. This result is in line with recent neurocognitive findings showing that it is not so much the availability of lexical representations but even more so the accessibility of these representations that predict success in orthographic decoding in typical and atypical readers (Boets et al., 2013). Apparently, the availability of lexical representations in temporal parts of the brain need to be accompanied by connections in the frontal part facilitating automated retrieval of phonological segments from memory. To conclude, the present findings highlight the importance of high-quality lexical representations. It should also be kept in mind that our confirmatory factor analysis showed phonological awareness to be highly related to the precursor measures of vocabulary breadth and depth and phonological coding, both tapping the quantity and quality of phonological representations in the mental lexicon. Our results thus seem to indicate that the availability of phonological representations can be seen as a necessary but not sufficient condition for the emergence of literacy to take place. In order to make the step from spoken language to literacy, children must be able to access fine-grained phonemic codes in their mental lexicon which can be assembled to graphemic codes.

The present study has as limitation in that lexical quality measures have only been measured in the beginning of children’s second kindergarten year. Another limitation is that context measures, such as children’s contact with literacy in home and school settings, have not been taken into account. In order to get a more complete account of the relationship between lexical quality and emergent literacy in kindergarten, there is a need of long-term longitudinal studies in which lexical quality measures and early literacy measures are documented in relation to children’s literacy environment.

To conclude, the present study shows that accessibility to fine-grained phonological representations, as measured by phonological awareness and lexical retrieval can be seen as the essential lexical quality measures predicting the emergence of literacy in kindergarten, even after controlling for vocabulary, phonological coding abilities and verbal working memory. For educators, it is important to highlight the transition that children at kindergarten level need to make from implicit to explicit phonological abilities in order to make the step from oral language to literacy.
